# An investigation of the determinants of quality of life in adolescents and young adults with Down syndrome

**DOI:** 10.1371/journal.pone.0197394

**Published:** 2018-06-13

**Authors:** Fatma Haddad, Jenny Bourke, Kingsley Wong, Helen Leonard

**Affiliations:** 1 University of Western Australia, Perth Western Australia, Australia; 2 Telethon Kids Institute, University of Western Australia, Perth Western Australia, Australia; Universita degli Studi di Perugia, ITALY

## Abstract

**Background:**

Young people with Down syndrome experience varying abilities in activities of daily living, cognitive functioning, behaviour and social skills. The aim of this research was to investigate, from a carer’s perspective, the factors that influenced the quality of life of these young people.

**Methods:**

Families of young people with Down syndrome (*n* = 197), aged 16–31 years, living in Western Australia, took part in a questionnaire study regarding young person daily functioning, family characteristics, medical background and quality of life measured by the Kidscreen 27-item scale. Kidscreen-10 total score was used as an outcome in the investigation of determinants with higher scores indicating better quality of life.

**Results:**

After adjustment for confounders including carer’s mental health measured by the Depression and Anxiety Scale (DASS), global impact of illness as well as impact of mental health and bowel conditions were all negatively associated with the young person’s quality of life. Young people who had three or more friends had better quality of life than those with no friends. Scores were lower (reflecting poor quality of life) in individuals who had more behavioural problems but these relationships were attenuated after adjustment for confounders and DASS.

**Conclusions:**

Overall, our findings revealed that quality of life of young people with Down syndrome was most negatively associated with burden of medical conditions, but also with lack of friendships. We were somewhat surprised to find the effect of medical problems on quality of life persisting into adolescence and adulthood where in general the burden of medical comorbidities is much less than in childhood.

## Introduction

The concept of quality of life (QOL) is one that broadly encompasses aspects of health and wellbeing. It can be considered as a “complete state of physical, mental, and social wellbeing and not merely the absence of infirmity”, as defined by the WHO [1], or simply as the subjective feelings of satisfaction and wellbeing which occur in relation to life experiences [2]. Generally, quality of life can be a difficult entity to measure, as each person attains unique perceptions of their overall quality of life, which is influenced by cultural contexts, previous experiences, personal values, and aspirations[3].

Down syndrome, also known as trisomy 21, is the commonest known medical cause of intellectual disability [4,5], occurring at a rate of approximately 1 in 1000 live births [6]. Individuals with Down syndrome have varying degrees of impairment, with many experiencing difficulties in communication and understanding [7]. They may also experience emotional and behavioural challenges [8–10] which may be associated with activity limitations in the areas of social interactions and community skills [11]. As a consequence of communication and cognitive limitations, assessing QOL can be difficult in this population. Previous research involving self-report by young people with intellectual disability has been subject to bias with concern about study reliability and validity [12,13]. However, within a human rights framework, these individuals have the right to attain personal goals and to experience a QOL equivalent to those who do not have a disability and to be able to access suitable, evidence-based services that support them in these goals[14]. Therefore it is important not only to be able to better measure QOL in this population but also to investigate the factors and relationships which impact on it.

Young people with Down syndrome often experience a number of health-related issues, which may affect overall wellbeing. These issues can transcend multiple physical systems and include cardiovascular, gastrointestinal, respiratory, and endocrine abnormalities [15,16]. Like others with intellectual disability but to a lesser extent [17], people with Down syndrome are more likely than their typical peers to experience emotional and behavioural disturbance [18] which can adversely impact on opportunities for social engagement and community participation. Those with higher levels of everyday functioning are more likely to be in employment as are those with fewer behavioural problems [19]. This population may also be at particular risk of developing depressive symptomatology into adult life [20]. The characteristic facial features associated with Down syndrome make it a visible disability that can both impair social acceptance and promote isolation [21]. Despite this, the “Down syndrome Advantage” has been proposed, suggesting that families with a child with Down syndrome experience less stress and greater rewards than families with a child with intellectual disability of another cause [22].

While there is a considerable body of literature focussing on the wellbeing of their family, including mothers and siblings [23–27], there has been comparatively less research on the wellbeing of the young people with Down syndrome themselves. This highlights the importance of investigating this construct and the underlying influences in this population. Thus, the aim of the research was to investigate determinants of health-related quality of life of young people with Down syndrome, with a focus on the identification of risk and protective factors pertaining not only to the individuals themselves but also to their family environment.

## Methods

Recruitment for this study was from the Western Australian population-based Down syndrome database. The database was first established in 1997 with families of school-aged children identified through the state-wide Disability Services Commission and later the population-based Intellectual Disability Exploring Answers (IDEA) database [28]. In 2004, families of young people with Down syndrome, aged 0–25 years, were invited to participate in an expanded study[25]. In 2009 and 2011 families of those born prior to 1994 were invited to complete further questionnaires in a study which focussed on issues related to transition into adulthood. The current study used data collected in 2011 from caregivers of the young people, with the majority of informants being the young persons’ biological mothers.

The carer-report questionnaire was developed with both consumer and stakeholder input and included two components. The first section was about the individual with Down syndrome including health, functioning, daily occupations, social relationships, quality of life and behaviour, and the second section was about the quality of life and health of the family. Ethical approval was obtained from the Ethics Committee of the Women’s and Children’s Health Services in Western Australia.

### Measures

The outcome variable for this study was health-related quality of life of young people with Down syndrome as estimated by caregiver completion of the Kidscreen-27 Parent Proxy Report questionnaire [25] allowing calculation of the five individual domain scores. Individual domain sub-scores were only calculated if there were no missing items. In order to obtain a composite score the 10 items forming the Kidscreen-10 measure were used with a maximum of one missing value allowed and imputed. A Rasch-scaled [29] single sum score was then transformed into T-values with a mean of 50 and a standard deviation of approximately 10, according to the manual.

Personal sociodemographic information included gender and age (16–18, 19–22, 23–31 years). Parental sociodemographic factors included maternal age (38–45, 46–55, 56 and older years) and paternal age (42–55, 56–65, 66 and older years), maternal and paternal highest level of education (primary school, high school, advanced diploma, Bachelor or higher degree), and maternal and paternal work status (full time work, part-time work, not working).

Environmental or family factors such as living region (major city, inner regional, and outer regional and remote), annual family income (AU$31,199 or less, AU$31,200—AU$51,999, AU$52,000–77,999, and AU$78,000 or higher), savings and carer’s mental health were measured. Savings was categorised as: 1) No savings, includes: a. we are spending money we haven’t got; b. we have just enough money to get us through to the next pay day; c. there’s some money left over each week but we just spend it, 2) We can save a bit every now and again, and 3) We can save a lot. Carer mental health was measured using the Depression Anxiety and Stress Scales (DASS 21), a screening tool with three sub-scales that assess symptoms in depression, anxiety, and stress and with validity in both clinical and community settings [30,31].

Personal social factors included number of friends, meeting of needs, day occupation, functioning, and behaviour. The number of friends was categorised into 3 groups; (no friends, 1–2 friends, and 3 or more friends). Needs being met was a binary measure (yes/no) in response to the question “Do you feel that your son’s/daughter’s needs are being met under the current day activity arrangements?” Day occupation was categorised in a descending hierarchy as: 1) in open work environment (must not be in school), 2) in a sheltered service environment (must not be in open work environment and must not be in school), and 3) post secondary classes, alternatives to employment, and not working (must not be in a sheltered service environment, in open work environment, or in school). The young person’s functioning in activities of daily living (ADL) was measured using the Index of Social Competence (ISC)[32]. The ISC has 3 subscales, community, self-care and communication skills. Each subdomain is examined individually and a total score of all three subscales (i.e. functioning total) was also assessed for this research. Behaviour was measured using the Developmental Behaviour Checklist Adult version (DBC-A) [9,33]. The DBC, which has been found to be a valid and reliable tool in measuring psychopathology, was developed specifically for people with intellectual and developmental disability [9,33]. It is a 107-item checklist, with each behavioural description scored on a 3-point Likert scale; with 0 being “not true as far as you know”, 1 being “somewhat or sometimes true”, and 2 being “very true or often true”. The DBC provides measures of overall behavioural and emotional disturbance (Total Behaviour Problems Score [TBPS]) and 5 subscale scores derived from factor analysis [9]. The TBPS (referred to as “behaviour” throughout the study) has been found, in instrument validation studies, to be strongly associated with child psychiatrists’ ratings of severity of psychopathology, i.e. the higher the score the higher the likelihood of significantly marked emotional and behavioural disturbance [34]. Behaviour (i.e. TBPS score), initially a continuous variable, was treated as a binary variable representing scores of <46 and > = 46, as a score of 46 and over is consistent with the presence of a psychiatric disorder [9].

Personal medically related factors included body mass index (BMI) categorised as healthy weight (<25), overweight (25–30) or obese (>30), weekly physical activity (0–2 days, 3–4 days, and 5 or more days), 12 monthly GP or psychologist visit for mental health reasons (yes, no), and medical impact. Medical impact was assessed using 13 questions asking about the impact of a range of conditions including heart, bowel, skin, and mental health. Each medical condition was scored based on caregiver’s rating of impact on daily life (0 = none, 1 = minor, 2 = moderate, or 3 = major). For each individual, a total score was calculated by summing the individual item scores. Global impact was then categorised into 4 subgroups (0, 1–4, 5–8, 9 and above). The higher the global impact, the higher the burden of disease. Furthermore, the impact of several specific medical conditions, including mental health, bowel conditions, skin conditions, and hearing loss, was also calculated. Results for these specific conditions were divided into two categories based on impact: 1) none to minor impact, and 2) moderate to major impact.

### Statistical analysis

Descriptive statistics were used to summarise the characteristics of the cohort, including those who did or did not meet the inclusion criteria of Kidscreen completion and the overall population of the 2011 study (i.e. 197 participants). Each Kidscreen-27 domain sub-score was only calculated if it had no missing items and Kidscreen-10 total score allowed a maximum of one missing item. Simple linear regression was used to examine the univariate relationship between Kidscreen-10 and the sociodemographic, family, social and medically related factors. Multiple linear regression was used to examine the association between Kidscreen-10 and living region, savings, child occupation, friendships, behaviours, physical activity, and medically related variables allowing adjustment for confounders including young person’s age and gender, parental factors (including maternal and paternal age, maternal and paternal education, and maternal and paternal occupation), and young person’s functioning. When adjusting for confounders, we examined the relationship once with the DASS and once without the DASS, in order to observe any change that may be affected by carer’s mental health and to explore any relationships that the variable may impact. Adjusted estimates and their 95% confidence intervals, and the P-value were reported. Estimated means of Kidscreen-10 were predicted by using Stata’s post-estimation command “margins” after multiple regression models were fitted. STATA 14.2 was used for all analyses.

The relative importance of factors affecting QOL, both positively and negatively after adjustment for DASS, have been graphically displayed.

## Results

Questionnaires were administered to 232 families of adolescents and young people aged 16 years and above and responses were received from 197 (84.9%). Of those, 175 (88.8%) met the criteria for Kidscreen-10 completion. A summary of those included in the study, those not included, and total number of participants is presented in Table 1.

**Table 1:** Characteristics of 197 young people with Down syndrome, by Kidscreen-10 completion status.

	Kidscreen-10			
	Completed (N = 175)		Did not complete (N = 22)	
**Personal sociodemographic factors**				
**Gender, n (%)**				
Male	97	(55.4)	13	(59.1)
Female	78	(44.6)	9	(40.9)
**Age group, n (%)**				
16–18 years	25	(14.3)	5	(22.7)
19–22 years	54	(30.9)	4	(18.2)
23–31 years	96	(54.9)	13	(59.1)
**Parent sociodemographic factors**				
**Mother’s age, n (%)**				
38–45	13	(7.4)	2	(9.1)
46–55	68	(38.9)	6	(27.3)
56 and older	90	(51.4)	14	(63.6)
Missing	4	(2.3)	0	(0)
**Father’s age, n (%)**				
42–55	69	(39.4)	7	(31.8)
56–65	63	(36.0)	8	(36.4)
66 and older	25	(14.3)	4	(18.2)
Missing	18	(10.3)	3	(13.6)
**Mother’s occupation, n (%)**				
Full-time work	36	(20.6)	7	(31.8)
Part-time work	65	(37.1)	6	(27.3)
Not working	68	(38.9)	9	(40.9)
Missing	6	(3.4)	0	
**Father’s occupation, n (%)**				
Full-time work	85	(48.6)	7	(31.8)
Part-time work	16	(9.1)	1	(4.6)
Not working	36	(20.6)	7	(31.8)
Missing	38	(21.7)	7	(31.8)
**Mother’s education**^**a**^**, n (%)**				
Primary school	40	(22.8)	7	(31.8)
Completed high school	38	(21.7)	3	(13.6)
Advanced diploma	32	(18.3)	3	(13.6)
Bachelor degree	60	(34.3)	8	(36.4)
Missing	5	(2.9)	1	(4.6)
**Father’s education**^**a**^**, n (%)**				
Primary school	24	(13.7)	2	(9.1)
Completed high school	25	(14.3)	1	(4.6)
Advanced diploma	48	(27.4)	7	(31.8)
Bachelor degree	58	(33.1)	6	(27.3)
Missing	20	(11.4)	6	(27.3)
**Family factors**				
**Living region, n (%)**				
Major cities of Australia	128	(73.1)	16	(72.7)
Inner regional Australia	20	(11.4)	4	(18.2)
Outer regional and remote	26	(14.9)	2	(9.1)
Missing	1	(0.6)	0	(0)
**Annual family income, n (%)**				
Less than AU$31,199	30	(17.1)	6	(27.3)
Between AU$31,200 and AU$51,999	28	(16.0)	2	(9.1)
Between AU$52,000 and AU$71,999	23	(13.1)	0	(0)
Above AU$72,000	77	(44.0)	5	(22.7)
Missing	17	(9.7)	9	(40.9)
**Savings, n (%)**				
No savings	44	(25.1)	8	(36.4)
We can save a bit	92	(52.6)	6	(27.3)
We can save a lot	35	(20.0)	2	(9.1)
Missing	4	(2.3)	6	(27.3)
**Carer’s emotional state (DASS), n mean (SD) range**				
Depression of all mothers	139	5.8 (7.0) 0–34	11	6.7 (6.6) 0–20
Depression of all carers	162	5.7 (6.8) 0–34	12	6.3 (6.5) 0–20
Anxiety of all mothers	139	3.4 (5.7) 0–28	11	4 (6.7) 0–20
Anxiety of all carers	162	3.5 (5.5) 0–28	12	4 (6.4) 0–20
Stress of all mothers	139	8.4 (8.0) 0–40	11	8.4 (6.1) 0–20
Stress of all carers	162	8.3 (7.7) 0–40	12	8.8 (6.0) 0–20
**Personal social factors**				
**Day occupation**^**b**^**, n (%)**				
Open work environment	36	(20.6)	3	(13.6)
In a sheltered workshop	76	(43.4)	6	(27.3)
Post-secondary classes	45	(25.7)	7	(31.8)
Missing	18	(10.3)	6	(27.3)
**Number of friends, n (%)**				
No friends	35	(20.0)	5	(22.7)
1–2 friends	77	(44.0)	7	(31.8)
3 or more friends	63	(36.0)	4	(18.2)
Missing	0	(0)	6	(27.3)
**Needs are met**^**c**^**, n (%)**				
Yes	114	(65.1)	11	(50.0)
No	47	(26.9)	5	(22.7)
Missing	14	(8.0)	6	(27.3)
**Behaviour, n (%)**				
<46	137	(78.3)	12	(54.6)
46 and higher	36	(20.6)	2	(9.1)
Missing	2	(1.1)	8	(36.4)
**Functioning in activities of daily living, n mean (SD) range**				
Self care	175	20.2 (4.0) 7–26	21	17.5 (5.0) 7–24
Community skills	175	11.4 (4.2) 4–19	21	8.4 (4.1) 4–16
Communication skills	175	5.8 (1.5) 2–8	21	4.7 (1.7) 2–8
Total	175	37.4 (8.6) 13–52	21	30.6 (9.6) 13–47
**Personal medically related factors**				
**BMI, n (%)**				
Healthy weight (<25)	37	(21.1)	5	(22.7)
Overweight (25–30)	38	(21.7)	1	(4.6)
Obese (>30)	56	(32.0)	5	(22.7)
Missing	44	(25.1)	0	(0)
**Physical activity per week, n (%)**				
0–2 days	56	(32.0)	5	(22.7)
3–4 days	56	(32.0)	1	(4.6)
5 days or more	57	(32.6)	8	(36.4)
Missing	6	(3.4)	8	(36.4)
**Mental health visits, n (%)**				
No	147	(84.0)	12	(54.6)
Yes	26	(14.9)	5	(22.7)
Missing	2	(1.1)	5	(22.7)
**Global impact of illness, n (%)**				
0	27	(15.4)	7	(31.8)
1–4	58	(33.1)	4	(18.2)
5–8	39	(22.3)	4	(18.2)
9 and above	51	(29.1)	7	(31.8)
**Impact of mental health illnesses, n (%)**				
None to minor	149	(85.1)	20	(90.9)
Moderate to major	26	(14.9)	2	(9.1)
**Impact of skin conditions, n (%)**				
None to minor	144	(82.3)	21	(95.5)
Moderate to major	31	(17.7)	1	(4.6)
**Impact of bowel conditions, n (%)**				
None to minor	151	(86.3)	19	(86.4)
Moderate to major	324	(13.7)	3	(13.6)
**Impact of hearing loss, n (%)**				
None to minor	145	(82.9)	19	(86.4)
Moderate to major	30	(17.1)	3	(13.6)

^a^ Grouped and categorised as primary school and some high school, completed high school Year 12 or equivalent, advanced diploma, bachelor degree or graduate diploma or certificate and postgraduate degree (Masters or PHD).

^b^ Open work environment: must not be attending school. Post secondary classes: alternatives to employment, and not working.

^c^ Needs are met under the current day activity arrangements

Among families with complete Kidscreen data, more young people were male (55.4%), aged at or above 23 years (54.9%) and living in the metropolitan area (73.1%). They were most likely to work primarily in a sheltered workshop (43.4%) and fewer participated in post secondary education (25.7%) or worked in an open environment (20.6%). The majority (80.0%) had at least one friend, a similar proportion (78.3%) did not meet the DBC clinical cut-off for a behaviour disorder and caregivers of nearly two thirds (65.1%) felt that their needs were met. The mean total score of functioning in activities of daily living was 37.4 (SD 8.6). In terms of health, close to a third of young people were obese (32.0%) and most (84.0%) had never visited their health professionals for mental health reasons. For just over a half (51.4%) medical problems impacted on their daily life, as reflected by the global and specific measures. Regarding the families of young people, close to half (48.6%) of the fathers worked full-time and most (60.5%) had post secondary school education, whilst the mothers were either part-time workers (37.1%) or not working (38.9%) despite more than half (52.6%) having a trade qualification or bachelor degree. Most of the families earned more than AU$52,000 per year (57.1%) and were able to save a bit (52.6%) or a lot (20.0%). The carer’s emotional state subscale scores for depression, anxiety and stress of all carers were 5.7 (SD 6.8), 3.5 (SD 5.5) and 8.3 (SD 7.7) respectively.

The mean Kidscreen-10 score was 42.0 (SD 8.4, range 26.1–87.9). The mean Kidscreen-27 scores for the five domains were Physical Well-being (38.1 (SD 8.1)), Psychological Well-being (45.6 (SD 9.4)), Autonomy and Parent Relations (50.6 (SD 11)), Social Support and Peers (35.8 (SD 12.7)) and School Environment (48.0 (SD 8.3)) (Table 2).

**Table 2:** Kidscreen-27 Domain T- scores of 197 young people with Down syndrome, by Kidscreen-10 completion status.

	Kidscreen-10			
Kidscreen-27 Domain	Completed (N = 175)		Did not complete (N = 22)	
	n	mean (SD) range	n	mean (SD) range
Physical well-being	170	38.1 (8.1) 23.1–71.2	7	36.0 (7.5) 26.3–49.5
Psychological well-being (Mood)	163	45.6 (9.4) 23.3–76.4	7	45.0 (13.9) 23.3.-63.1
Autonomy and parent relation (Family)	153	50.6 (11.0) 27.2–79.1	2	45.5 (11.3) 37.5–53.4
Social support and peers (Friends)	159	35.8 (12.7) 9.3–70.3	5	36.3 (8.3) 27.5–49.1
School environment (Daily activity)	170	48.0 (8.3) 26.1–70.7	8	48.4 (4.8) 41.2–55.4

SD, standard deviation

Univariate relationships between Kidscreen-10 and young person sociodemographic, social and medically related factors are presented in Table 3.

**Table 3:** Univariate relationships between Kidscreen-10 and sociodemographic, social and medically related factors in 175 young people with Down syndrome.

Factors	n	Mean (SD)	Range	Coefficient (95% CI)	P-value
**Gender**					
Male	97	42.1 (8.2)	26.1–70.4	Reference	
Female	78	41.8 (8.6)	29.8–87.9	-0.2 (-2.8,2.3)	0.85
**Age group (yrs)**					
16–18	25	40.1 (8.0)	26.1–68.0	Reference	
19–22	54	41.7 (9.6)	29.8–87.9	1.6 (-2.4,5.6)	0.43
23–31	96	42.6 (7.7)	29.8–70.4	2.4 (-1.3,6.2)	0.20
**Day occupation**^**a**^ **(n = 157)**					
Open work environment	36	43.0 (7.5)	29.8–59.9	Reference	
In a sheltered service environment	76	42.7 (8.4)	31.0–87.9	-0.4 (-3.7,2.9)	0.82
Post secondary classes	45	39.9 (8.4)	29.8–70.4	-3.2 (-6.8,0.5)	0.09
**Needs met**^**b**^ **(n = 161)**					
Yes	114	42.7 (8.4)	26.1–87.9	Reference	
No	47	39.9 (7.9)	29.8–66.2	-2.8 (-5.6,0.1)	0.06
**Number of friends**					
No friends	35	37.1 (5.7)	26.1–50.6	Reference	
1–2 close friends	77	41.6 (7.4)	29.8–70.4	4.5 (1.3,7.6)	0.01
3 or more friends	63	45.1 (9.5)	32.2–87.9	8.0 (4.7,11.3)	<0.01
**Behaviour (n = 173)**					
<46	137	43.3 (8.5)	29.8–87.9	Reference	
46 and higher	36	37.0 (6.1)	26.1–57.3	-6.2 (-9.2,-3.3)	<0.01
**Functioning in activities in daily living**					
Self care	175	20.2 (4.0)^c^	7–26^c^	0.7 (0.4,1.0)	<0.01
Community skills	175	11.4 (4.2)^c^	4–19^c^	0.5 (0.2,0.8)	<0.01
Communication skills	175	5.8 (1.5)^c^	2–8^c^	1.5 (0.7,2.3)	<0.01
Total	175	37.4 (8.6)^c^	13–52^c^	0.3 (0.2,0.5)	<0.01
**BMI (n = 131)**					
Healthy weight (<25)	37	42.3 (7.5)	29.8–59.9	Reference	
Overweight (25–30)	38	43.0 (8.1)	31.0–68.0	0.7 (-2.9,4.4)	0.70
Obese (>30)	56	41.1 (8.4)	26.1–70.4	-1.2 (-4.6,2.2)	0.48
**Physical activity per week (n = 169)**					
0–2 days	56	40.2 (8.1)	26.1–66.2	Reference	-
3–4 days	56	44.3 (9.7)	29.8–87.9	4.0 (0.9,7.1)	0.01
5 or more days	57	42.0 (6.9)	32.2–68.0	1.8 (-1.3,4.9)	0.25
**Mental Health visits**^**d**^ **(n = 173)**					
No	147	42.8 (8.7)	29.8–87.9	Reference	
Yes	26	37.7 (5.3)	26.1–46.7	-5.1 (-8.6,-1.6)	<0.01
**Global impact of illness**					
0	27	48.1 (11.5)	33.5–87.9	Reference	
1–4	58	42.4 (7.5)	29.8–66.2	-5.6 (-9.2,-2.0)	<0.01
5–8	39	42.0 (8.0)	31.0–70.4	-6.1 (-9.9,-2.2)	<0.01
9 and above	51	38.2 (5.5)	26.1–50.6	-9.8 (-13.5,-6.1)	<0.01
**Impact of mental health conditions**^**e**^					
None to minor	149	42.9 (8.4)	29.8–87.9	Reference	
Moderate to major	26	36.9 (6.1)	26.1–50.6	-5.9 (-9.3,-2.5)	<0.01
**Impact of skin conditions**^**f**^					
None to minor	144	42.8 (8.6)	29.8–87.9	Reference	
Moderate to major	31	38.3 (6.3)	26.1–55.8	-4.5 (-7.7,-1.3)	<0.01
**Impact of bowel conditions**^**g**^					
None to minor	151	42.5 (8.5)	29.8–87.9	Reference	-
Moderate to major	24	38.6 (6.5)	26.1–57.3	-3.9 (-7.5,-0.3)	0.03
**Impact of hearing loss**					
None to minor	145	42.7 (8.5)	29.8–87.9	Reference	-
Moderate to major	30	38.3 (6.6)	26.1–59.9	-4.5 (-7.8,-1.2)	<0.01

^a^ Open work environment: must not be attending school). Post secondary classes: alternatives to employment, and not working

^b^ Needs are met under current day activity arrangements

^c^ Functioning in activities of daily living score

^d^ Visits to either psychologist or doctor

^e^Depression, anxiety, and other mental health

^f^ Psoriasis and eczema

^g^ Constipation, coeliac, and other bowel conditions

CI, confidence interval; SD, standard deviation

Univariate relationships between Kidscreen-10 and parental and family factors are presented in Table 4.

**Table 4:** Univariate relationships between Kidscreen-10 T-score and parental sociodemographic and family factors in 175 young people with Down syndrome.

Factors	n	Mean (SD)	Range	Coefficient (95% CI)	P-value
**Maternal age (years) (n = 171)**					
38–45	13	38.3 (3.1)	33.0–45.0	Reference	
46–55	68	41.7 (7.3)	26.1–59.9	3.4 (-1.5,8.3)	0.18
56 and older	90	42.5 (9.3)	29.8–87.9	4.2 (-0.6,9.0)	0.08
**Paternal age (years) (n = 157)**					
42–55	69	40.8 (6.6)	26.1–57.3	Reference	
56–65	63	41.2 (7.1)	29.8–59.9	0.4 (-2.4,3.2)	0.80
66 and older	25	45.8 (13.1)	31.0–87.9	5.0 (1.2,8.7)	0.01
**Maternal work status (n = 169)**					
Full time work	36	42.3 (10.8)	29.8–87.9	Reference	
Part-time work	65	41.6 (6.5)	26.1–59.9	-0.7 (-4.1,2.7)	0.69
Not working	68	42.0 (8.3)	29.8–70.4	-0.2 (-3.6,3.2)	0.89
**Paternal work status (n = 137)**					
Full time work	85	42.4 (8.4)	29.8–87.9	Reference	
Part-time work	16	41.8 (8.3)	29.8–66.2	-0.5 (-4.9,3.9)	0.81
Not working	36	40.8 (7.5)	29.8–57.3	-1.5 (-4.7,1.7)	0.35
**Maternal education (n = 170)**					
Primary school	40	42.0 (7.7)	29.8–66.2	Reference	
High school	38	42.0 (6.8)	29.8–57.3	0.0 (-3.7,3.7)	0.99
Advanced diploma	32	41.7 (10.8)	32.2–87.9	-0.3 (-4.2,3.6)	0.87
Bachelor degree or higher	60	41.8 (8.1)	26.1–70.4	-0.2 (-3.5,3.2)	0.92
**Paternal education (n = 155)**					
Primary school	24	40.4 (7.5)	26.1–57.3	Reference	
High school	25	40.5 (6.8)	29.8–57.3	0.1 (-4.4,4.7)	0.95
Advanced diploma	48	42.1 (9.2)	29.8–87.9	1.7 (-2.3,5.7)	0.41
Bachelor degree or higher	58	42.1 (7.9)	31.0–70.4	1.7 (-2.2,5.6)	0.39
**Living region (n = 174)**					
Major cities of Australia	128	42.5 (8.7)	29.8–87.9	Reference	
Inner regional Australia	20	42.6 (8.9)	26.1–68.0	0.1 (-3.9,4.1)	0.96
Outer regional and remote Australia	26	39.3 (6.1)	29.8–57.3	-3.2 (-6.8,0.4)	0.08
**Annual Family income (n = 158)**					
Less than $31,199	30	41.8 (9.9)	26.1–68.0	Reference	
Between $31,200 and 51,999	28	38.8 (7.7)	29.8–59.9	-3.1 (-7.0,0.9)	0.13
Between 52,000 and 77,999	23	39.7 (4.6)	29.8–48.6	-2.2 (-6.4,2.0)	0.30
Above 78,000	77	43.0 (7.3)	31.0–70.4	1.1 (-2.1,4.4)	0.49
**Savings (n = 171)**					
No savings	44	37.8 (7.2)	26.1–68.0	Reference	
We can save a bit every now and again	92	43.2 (8.6)	29.8–87.9	5.3 (2.4,8.3)	<0.01
We can save a lot	35	44.0 (8.1)	31.0–70.4	6.2 (2.5,9.8)	<0.01
**DASS**					
Anxiety of all mother	139	3.4 (5.7)^a^	0–28^a^	-0.3 (-0.5,-0.1)	<0.01
Anxiety of all caregivers	162	3.5 (5.5)^a^	0–28^a^	-0.3 (-0.5,-0.1)	<0.01
Depression of mother	139	5.8 (7.0)^a^	0–34^a^	-0.4 (-0.6,-0.2)	<0.01
Depression of all caregivers	162	5.7 (6.8)^a^	0–34^a^	-0.4 (-0.6,-0.3)	<0.01
Stress of mother	139	8.4 (8.0)^a^	0–40^a^	-0.3 (-0.5,-0.2)	<0.01
Stress of all caregivers	162	8.3 (7.7)^a^	0–40^a^	-0.4 (-0.5,-0.2)	<0.01

^a^ DASS score

CI, confidence interval; SD, standard deviation

Adjusted assocations between Kidscreen-10 and relevant factors with or without adjusting for DASS are shown in Table 5.

**Table 5:** Multiple regression analysis for Kidscreen-10.

	Without adjusting for DASS			With adjusting for DASS		
	Adjusted coefficient (95% CI)	P-value	Estimated mean^a^ (95% CI)	Adjusted coefficient (95% CI)	P-value	Estimated mean^a^ (95% CI)
**Personal sociodemographic and social factors**						
**Friends**	n = 121			n = 118		
No friends	Reference		38.4 (35.1,41.7)	Reference		38.1 (34.9,41.4)
1–2 friends	2.6 (-1.5,6.6)	0.21	41.0 (38.9,43.2)	3.2 (-0.7,7.2)	0.11	41.4 (39.2,43.5)
3 or more friends	6.0 (1.6,10.4)	0.01	44.4 (41.9,46.9)	6.1 (1.9,10.4)	<0.01	44.3 (41.9,46.7)
**Day occupation**^**b**^	n = 108			n = 105		
Open work environment	Reference		40.3 (36.7,43.9)	Reference		39.9 (36.3,43.5)
In a sheltered service environment	3.2 (-1.3,7.6)	0.16	43.5 (41.2,45.8)	4.0 (-0.4,8.4)	0.08	43.9 (41.7,46.1)
Post secondary classes	-0.9 (-6.2,4.4)	0.75	39.5 (36.2,42.7)	-0.7 (-5.8,4.5)	0.79	39.2 (36.1,42.4)
**Behaviour**	n = 121			n = 118		
<46	Reference		42.5 (40.8,44.2)	Reference		42.1 (40.4,43.9)
46 and higher	-3.9 (-8.0,0.2)	0.07	38.7 (35.2,42.1)	-1.7 (-6.4,3.0)	0.47	40.4 (36.6,44.3)
**Personal medically related factors**						
**Physical activity**	n = 117			n = 114		
0–2 days	Reference		41.0 (38.4,43.6)	Reference		41.2 (38.7,43.8)
3–4 days	3.4 (-0.4,7.2)	0.08	44.4 (41.7,47.1)	2.8 (-1.0,6.7)	0.15	44.0 (41.3,46.8)
5 or more days	-0.9 (-4.7,2.8)	0.62	40.0 (37.3,42.8)	-0.8 (-4.6,3.0)	0.67	40.4 (37.6,43.2)
**Global impact of illness**	n = 121			n = 118		
0	Reference		48.5 (44.5,52.4)	Reference		48.7 (44.7,52.8)
1–4	-6.0 (-10.7,-1.4)	0.01	42.5 (40.0,44.9)	-6.7 (-11.4,-2.1)	0.01	42.0 (39.6,44.4)
5–8	-7.0 (-12.0,-1.9)	<0.01	41.5 (38.5,44.5)	-6.6 (-11.7,-1.5)	0.01	42.1 (39.1,45.1)
9 and above	-10.2 (-15.1,-5.3)	<0.01	38.2 (35.6,40.9)	-10.1 (-15.1,-5.1)	<0.01	38.6 (36.0,41.3)
**Impact of mental health conditions**^**c**^	n = 121			n = 118		
None to minor	Reference		42.7 (41.1,44.2)	Reference		42.4 (40.8,44.0)
Moderate to major	-5.5 (-9.6,-1.5)	<0.01	37.1 (33.5,40.7)	-3.7 (-8.0,0.5)	0.09	38.7 (34.9,42.4)
**Bowel conditions**^**d**^	n = 121			n = 118		
None to minor	Reference		42.3 (40.7,43.8)	Reference		42.3 (40.8,43.8)
Moderate to major	-4.1 (-8.4,0.1)	0.06	38.1 (34.3,42.0)	-3.9 (-8.1,0.4)	0.07	38.4 (34.6,42.3)
**Skin conditions**^**e**^	n = 121			n = 118		
None to minor	Reference		42.0 (40.3,43.6)	Reference		42.2 (40.6,43.8)
Moderate to major	-1.7 (-5.8,2.5)	0.42	40.2 (36.6,44.0)	-2.5 (-6.7,1.6)	0.23	39.6 (36.0,43.3)
**Hearing loss**	n = 121			n = 118		
None to minor	Reference		42.2 (40.7,43.8)	Reference		42.1 (40.6,43.7)
Moderate to major	-3.2 (-7.2,0.7)	0.11	39.0 (35.5,42.5)	-2.1 (-6.2,1.9)	0.30	40.0 (36.4,43.6)
**Parental sociodemographic and family factors**						
**Savings**	n = 119			n = 117		
No savings	Reference		38.3 (35.2,41.6)	Reference		39.1 (35.8,42.4)
We save a bit every now and again	4.2 (0.3,8.0)	0.03	42.5 (40.6,44.5)	3.2 (-0.8,7.2)	0.11	42.3 (40.3,44.3)
We save a lot	4.5 (-0.5,9.5)	0.08	42.9 (39.3,46.4)	4.0 (-1.0,8.9)	0.12	43.0 (39.6,46.5)
**Living region**	n = 121			n = 118		
Major cities of Australia	Reference		42.3 (40.7,44.0)	Reference		42.6 (40.9,44.2)
Inner regional Australia	-2.3 (-7.8,3.2)	0.41	40.1 (35.0,45.1)	-3.0 (-8.3,2.3)	0.27	39.6 (34.7,44.5)
Outer regional and remote Australia	-3.4 (-7.7,1.0)	0.13	39.0 (35.0,42.9)	-3.8 (-8.0,0.5)	0.09	38.8 (34.9,42.7)

adjusted for young person’s age and gender, parental sociodemographic factors (including maternal and paternal age, maternal and paternal education, and maternal and paternal occupation), and young person’s functioning of activities of daily living total score

^a^ estimated mean of Kidscreen-10 T-value predicted from the multiple linear regression models

^b^ Open work environment: must not be attending school). Post secondary classes: alternatives to employment, and not working

^c^ Depression, anxiety, and other mental health

^d^ Constipation, coeliac, and other bowel conditions

^e^ Psoriasis and eczema

CI, confidence interval

### Association between Kidscreen-10 and young person and family factors

#### Personal sociodemographic and social factors

The mean Kidscreen-10 scores were similar between males and females (coefficient [β] -0.2; 95% confidence interval [CI] -2.8,2.3; P = 0.85). Compared to the 16–18 years age group, individuals in the older age group (e.g. 23–31 years) had better QOL (23–31 years: β 2.4; 95% CI -1.3,6.2; P = 0.20) (Table 3). Similarly, the mean score of individuals who had 3 or more friends was higher than that of young people with no friends (β 8.0; 95% CI 4.7,11.3; P<0.01) (Table 3), and the effect remained after accounting for confounders without DASS (adjusted β 6.0; 95% CI 1.6,10.4; P = 0.01) or with DASS (adjusted β 6.1; 95% CI 1.9,10.4; P<0.01) (Table 5). Better functioning in activities in daily living was also associated with an improvement in QOL (β 0.3; 95% CI 0.2,0.5; P<0.01) (Table 3). Compared to their respective reference groups, lower mean Kidscreen-10 scores were detected in individuals who participated in post secondary education (β -3.2; 95% CI -6.8,0.5; P = 0.09), had unmet need (β -2.8; 95% CI -5.6,0.1; P = 0.06) or were considered to have more behavioural problems (β -6.2; 95% CI -9.2,-3.3; P = <0.01) (Table 3). After adjusting for confounders, there was minimal difference in mean Kidscreen-10 score between the post secondary education and open work environment group (without DASS: adjusted β -0.9; 95% CI -6.2,4.4; P = 0.75, with DASS: adjusted β -0.7; 95% CI -5.8,4.5; P = 0.79). However, the decline in QOL related to more behavioural problems and differed depending on adjustment for DASS (without DASS: adjusted β -3.9; 95% CI -8.0,0.2; P = 0.07, with DASS: adjusted β -1.7; 95% CI -6.4,3.0; P = 0.47) (Table 5).

#### Personal medically related factors

Young people with Down syndrome who were deemed obese appeared to have poorer QOL when compared with those with healthy weight (β -1.2; 95% CI -4.6,2.2; P = 0.48). On the other hand, compared to those who were inactive (0–2 days per week), individuals with physical activity of 3 or more days per week had a higher mean Kidscreen-10 score (3–4 days: β 4.0; 95% CI 0.9,7.1; P = 0.01, 5 or more days: β 1.8; 95% CI -1.3,4.9; P = 0.25). Improvement in QOL remained for the 3–4 days group, but not for the 5 or more days group, after adjusting for confounders with or without DASS. Mental health visits, global impact of illness as well as impact of specific medical conditions were all negatively associated with young people’s quality of life (Mental health visit: β -5.1; 95% CI -8.6,1.6; P<0.01, 9 or above global impact of illness: β -9.8; 95% CI -13.5,-6.1; P<0.01) (Table 3). Adjusting for confounders with or without DASS did not affect the negative relationships (Table 5).

#### Parental sociodemographic and family factors

The mean Kidscreen-10 scores were higher in the oldest parental age group than in the youngest age group (maternal 56 years and older: β 4.2; 95% CI -0.6,9.0; P = 0.08, paternal 66 years and older: β 5.0; 95% CI 1.2,8.7; P = 0.01) (Table 4). Offspring’s QOL was not affected by parental work status or education level. Poorer QOL was observed for those living in outer regional and remote regions of Western Australia (β -3.2; 95% CI -6.8,0.4; P = 0.08), and the association extended to families in inner regional areas after accounting for confounders without DASS (β -2.3; 95% CI -7.8,3.2; P = 0.41) or with DASS (β -3.0; 95% CI -8.3,2.3; P = 0.27). Compared to families in the lowest annual income (AU $31,199 or less) group, QOL in the offspring of those earning AU$78,000 or higher was minimally improved (β 1.1; 95% CI -2.1,4.4; P = 0.49). Similarly QOL scores for those whose families were able to save, either a bit or a lot, were higher than those who had no savings (saved a bit: β 5.3; 95% CI 2.4,8.3; P<0.01, saved a lot: β 6.2; 95% CI 2.5,9.8; P<0.01) (Table 4), but the magnitude of improvement slightly diminished after adjusting for confounders with or without DASS (Table 5). Either mother’s or carer’s mental health status was negatively associated with their children’s QOL (Table 4).

**Fig 1:**
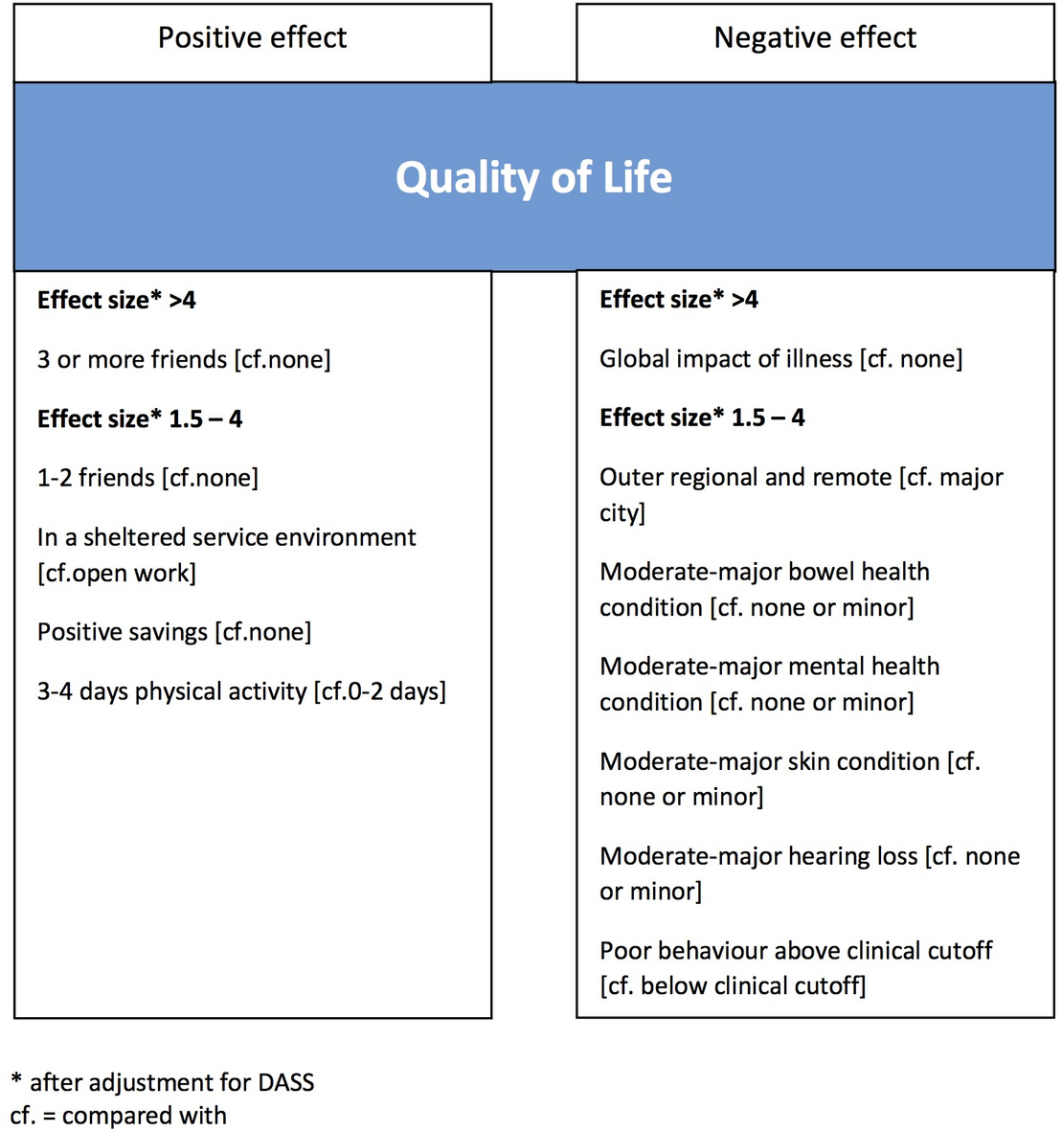
Relative importance of various factors affecting quality of life, both positively and negatively.

The relative importance of factors affecting QOL, both positively and negatively after adjustment for DASS, have been graphically displayed in Fig 1.

## Discussion

This study sought to evaluate quality of life in young people with Down syndrome and to identify its determinants including those related to the young person themselves as well as those related to their home environment. We found an overall mean Kidscreen-10 score of 42.0, considerably lower than population norms. Although, univariately, we found associations with a large number of individual factors, once adjusted and particularly when adjusted for mother’s mental state, we found that many of these associations were lost. What stood out however was the negative effect of high illness burden as well as the protective effect on QOL of having three or more friends.

A considerable strength of this study is that the data were collected from a population-based source and that there was a high response fraction to the administration of the questionnaire, thus ensuring representativeness of the underlying population. The comprehensive set of questions and instruments, including psychometrically validated tools such as the DBC, meant that there was a wide range of variables available for this investigation. Given that our study was based on parental report and that there has been previous concern that reporting on child impairment and quality of life can be mediated by maternal depression [35], we also took the important step of adjusting for carer mental health using the DASS in our multivariate analysis.

A limitation of the study relates to the fact that the assessment of QOL relied on data collected by proxy from parental reports, and that we obtained only one data source per participant. Data collection directly from young people with intellectual disability about their health, including mental health status presents numerous challenges and would not be amenable to a study design such as ours [12,13]. That said, a qualitative component exploring the personal definitions of what is considered good quality of life would bridge a gap in literature, and would empower young people with Down syndrome to express their own feelings.

To date there have only been two studies, both published very recently, examining QOL in Down syndrome in young people of a similar age range to ours [36,37]. Using Kidscreen-27, the earlier of the two compared individual domain scores with European normative data for the younger (12–18 year olds) component of their population while the second US study primarily involved a secondary analysis of PedsQL data collected in a study of body composition and metabolic risk in youth with Down syndrome [37]. In the European study the results for the young adult (aged 18–30 years) component of the population, were extremely consistent with our own findings, with lowest scores in the domains of physical wellbeing and social support while those for psychological wellbeing and school (possibly more appropriately categorised as work) environment were similar to population norms and those for autonomy and parent relations slightly higher. In contrast for the US study the scores for each of the domains apart from emotional functioning were lower than for a non-Down syndrome control group. In the first study data were collected on 90 individuals aged between 12 and 30 years from seven different countries and in the second US study from caregivers of 150 young people with Down syndrome, aged from 10–20 years. However, unlike our study neither study was derived from a population-based source and little detail was provided on recruitment or representativeness. In the US study using an Impact of Weight on Quality of Life measure lower scores were reported for obese compared with non-obese individuals irrespective of Down syndrome status. However in both studies there was an absence of data on other variables which would have permitted, as we have done, a comprehensive investigation of determinants of quality of life. The lower domain scores for physical wellbeing and social support seen in both studies were also consistent with the negative effects of disease burden and lack of friendships that we also identified. In another study, adults with Down syndrome aged 18–61 years completed an oral interview in which they provided responses to the Quality Metric Short Form-12 version 2 (SF-12v2) instrument [38]. Compared with a normative sample from the general population, these 60 adults with Down syndrome had higher than average quality of life scores, although the authors acknowledged that their sample was potentially healthier and not representative of the underlying population. Study representativeness is an essential element required for generalisation of findings. However, the only study we could find which considered this issue was a Dutch study restricted to 8 year old children [39], which had a response fraction of 63%, equivalent to around 50% of this birth cohort. Using the TNO-AZL Children’s Quality of Life (TACQOL) measure, an instrument developed in the Netherlands, children with Down syndrome were shown to have considerably poorer quality of life scores compared with population norms, particularly in the areas of gross motor skills, autonomy, social functioning, and cognitive functioning, although the latter might be expected, given that these children do have an intellectual disability. Although there is a growing body of research about the health-related quality of life of their parents and families, we could not find evidence of any further studies assessing quality of life in young people with Down syndrome and, specifically, none comparable with the age group in our study that considered the issue of representativeness.

Our findings paint a picture of worse QOL with increasing burden of illness. The relationship was observed specifically with bowel conditions and mental health problems as well as with our measure of the global impact of illness. Uniformly, a lower quality of life was reported for those with a higher burden of illness compared to those with no impact, with the effects persisting even after adjustment for mother’s mental health status. We were somewhat surprised to find the effect of medical problems on quality of life persisting into adolescence and adulthood where in general the burden of medical comorbidities is much less than in childhood [40]. In regard to mental illness our previous research has suggested that the presence of depressive symptomatology in adolescence/early adulthood might increase the risk of depressive illness occurring later in adulthood [20]. This highlights the importance of raising awareness among families and service providers and of routine screening for depressive symptoms [20].

Consistent with the findings from the international study where low scores were found in the “Social Support and Peers” domain [36], our other major finding related to the value of friendships and positive social relationships. Compared with those reported as having no friends, those with one to two friends had a higher Kidscreen score and it was much higher again in those with three or more friends. In the multivariate analysis after adjustment for other factors the relationship with three or more friends remained although that with one to two friends was attenuated. We previously found in an earlier study relating to school-aged children with Down syndrome that parents reported as many as a third of their children had no friends [41]. The proportion in this adolescent and young adult group with no friends was surprisingly slightly lower, although one might expect the reverse with friendships dropping off after school. In the previous study those with more friends were more likely to have better functional ability but in this study we still saw the relationship with quality of life after adjusting for functional ability.

We found that for young people with Down syndrome, QOL was reported as poorer the further away from the city (i.e. in outer regional and remote Australia) they lived. This is considered consistent with past research in the general population where those living further away from metropolitan areas have been shown to have poorer health outcomes and health risk factor profiles [42]. In our study, this relationship was observed in the univariate and multivariate analysis and did not attenuate after adjusting for carer mental health.

We had anticipated that young people with Down syndrome who participated in open employment would have better QOL than those in sheltered employment, given that such a relationship was found with family QOL[43] and since a higher level of functioning has been shown to be associated with increased likelihood of participation in open employment or training post-school [11]. However, this was not the case. In contrast we saw that better QOL was reported for those in sheltered employment. It is likely that sheltered employment provides more longterm security and stability for people with Down syndrome and possibly better opportunities for developing peer relationships. Further research into the impact of occupation (including frequency of work hours, type of work, interactions etc), in this group, is needed. This could assist policy makers and service providers with regards to occupational oriented services.

In our study, as might be expected from past research [39] behavioural issues were associated with a significant reduction in QOL in the univariate analysis but the effect was attenuated after adjustment for other factors, particularly caregiver mental health. Although previous research showed that approximately one quarter to one third of the young people with Down syndrome have significant behavior problems [44], we found a lower proportion would meet the clinical cut-off score for psychological caseness. Nevertheless, the development and chronicity of behaviour disorders, including risk factors, have been given less attention in the literature in people with intellectual disability [45]. Children with Down syndrome at risk of behavioural disorder in adult life may be identified in childhood and as a young person. Thus, there is a further need to target this area in future research, and appropriate interventions should be offered to reduce risk [46].

## Conclusion

This retrospective cohort study would appear to be the first to investigate a range of factors relating to QOL of young people with Down syndrome. Overall, our findings revealed that QOL of young people with Down syndrome was most negatively associated with burden of medical conditions, but also with lack of friendships. The negative effect of behavioural symptoms on quality of life was much reduced when other factors such as functioning were taken into account but the impact of a mental health condition was still associated with a reduced quality of life. Supporting young people with Down syndrome by educating service providers and advocating for a positive and judgment free environment is important in order to enhance quality of life and reduce stigma.
